# A Meteorological Table, by Dr. Higgins, of Brompton. From Dec. 20, 1804, to Jan. 22, 1805
*The Editors beg to acquaint Dr. Higgins, that his Meteorological Table for December has not been received; otherwise, it would most certainly have been inserted.


**Published:** 1805-02-01

**Authors:** 


					185
?4 Meteorological Table} by Dr. Higgins, of brompton.
From Dec. GO, 1804, to *JUn. <22, 1805.
Days
of the
Month
l hcrmometer,
Fahrenheit
Ccilte -
grade
Height of the Baro-
meter. Inches.
WEATHER.
Dec. 21
22
-*3
2 4
-25:
26
27
2ib
29
133^'
Us
,3o
I32 ;34
31 132
3? 28
31!
30
36
12
41 !43
|44 |45
139
831
9i32
3313s
14 35
15 38
*6 37
17
42
1832
19]
3i
|30
34
33
32
130
30
29
9
33
36
37
30
|4o
4o
l4i
30
29
31
7
35
40
32
36
!45
36
28
35
38
33
ioo.oo
I.II
2.22
ibel. 0
4.4-4
2.77
I.II
i 66
1.11
o 00
Ibel.
?-55
JI30.58I
24
129-95
I30.56
.08
29.96
130.52116?
29.93 14
30.13 13
130.16130.16
03129.97
.29.80! .80
88j .90
30.06,3 o. 06
.10 14
Ioo.oo
3-33
4-44
3-88
6.11
6.ii
7.22
3-88
, c-55
Ibel. 0
1.66
0.00
bel
!? Z Xj
6.11
4 44
3.88
6.66
7-77
4.44
3-33
6.66
3*33-
.27
?73
.65
?36
.19
.50
.52
.46
'3i
.19
?95
?94
?72
.46
?34
?73
.56
?30
.19
?51
?5S
.38
?35
.20
?95
.80
.65
-33
29.80'
.02
130.01
.06
.62
? 71
?42
.20
.30
?52'
?50! 14
,28[
?34
?34[
?94|
.81
?51
.19
14
0429.74 29 5T
?15
.16
29.31
.03!
?44'
30.00I
.0?
?00130.06
.2129.96
29.14I .02
'34
?53
.98
.80
[28.64
29-36
89
97
?77
130.22
7.9.44
04
?351 9
Fair.
Cloudy, snow in the evening.
Fair.
Fair.
Rain.
Rain and sleet.
Small rain.
Cloudy.
Cloudy.
Cloudy.
Fair.
Cloudy, rain in the evening.
Rain.
Cloudy.
Cloudy.
Cloudy.
Cloudy.
Rain in the morning,
Fair.
Cloudy.
Foggy.
Cloudy.
Cloudy, rain in the night.
Cloudy, rain in the evening.
Cloudy.
Showery.
Showery.
Cloudy, rain in the evening.
Cloudy.
Cloudy, rain in the evening.
Rain.
Cloudy, rain in the even'ng.
* The Editors beg to acquaint Dr. Iliggins, that his Meteorological
for December has not been received ; otherwise, it would most cei
been inserted.
Account

				

